# Typology of Work–Family Balance Among Middle–Aged and Older Japanese Adults

**DOI:** 10.3389/fpsyg.2022.751879

**Published:** 2022-03-16

**Authors:** Makiko Tomida, Yukiko Nishita, Chikako Tange, Takeshi Nakagawa, Rei Otsuka, Fujiko Ando, Hiroshi Shimokata

**Affiliations:** ^1^Department of Epidemiology of Aging, National Center for Geriatrics and Gerontology, Obu, Japan; ^2^Department of Social Science, National Center for Geriatrics and Gerontology, Obu, Japan; ^3^Department of Health and Medical Sciences, Aichi Shukutoku University, Nagakute, Japan; ^4^Graduate School of Nutritional Sciences, Nagoya University of Arts and Sciences, Nisshin, Japan

**Keywords:** work–family conflict, work–family facilitation, lifestyles, social support, mental health

## Abstract

This study explores the clusters of work–family balance (WFB) among Japanese middle-aged and older adults and clarifies the characteristics of the derived clusters. Data on working adults (*N* = 1,351; age range = 40–85 years) were drawn from a pool of participants in the National Institute for Longevity Sciences—Longitudinal Study of Aging. The WFB scale consists of subscales assessing work–family conflict (WFC) and work–family facilitation (WFF). First, a cluster analysis was performed using the WFB scale, and four clusters were extracted. Second, we examined associations between the four clusters and related variables such as demographic characteristics, work, family, and lifestyle factors, social support, and mental health. Our findings showed that the clusters included high-WFC/high-WFF, high-WFC/low-WFF, low-WFC/high-WFF, and low-WFC/low-WFF. Differences were found in related variables among the clusters. Specifically, those in the Low-WFC/High-WFF cluster had a good lifestyle, received the highest levels of social support, and had the fewest mental health issues. Our findings have implications for maintaining sufficient WFB and promoting positive mental health among workers.

## Introduction

Japan, with an aging rate of 28.1% reported in 2018, is a super-aged society where one in every four people is 65 years old or older (Japanese Cabinet Office, 2019). The proportion of older people in Japan’s labor force has continued to increase (from 4.9% in 1980 to 12.8% in 2018), with older people being highly motivated to work (Japanese Cabinet Office, 2019). It is crucial to identify ways to support workers across their lifespan as the country’s workforce ages (Baltes and Young, 2007).

Most empirical studies examining integrated frameworks of the work-nonwork interface are related to work–family balance (WFB). WFB is defined as “an overall appraisal of the extent to which individuals’ effectiveness and satisfaction in work and family roles, are consistent with their life values at a given point in time” (Greenhaus and Allen, 2011, p.174). The lack of work–family conflict (WFC) has been widely investigated within the context of WFB. WFC is defined as “a form of inter-role conflict in which role pressures from work and family domains are mutually incompatible in some respect” (Greenhaus and Beutell, 1985, p. 77). Other studies have called for a balanced approach recognizing the positive effects of combining work and family roles (e.g., Greenhaus and Powell, 2006). The concept of work–family facilitation (WFF) captures these positive effects. WFF occurs when “by virtue of participation in one role (e.g., work), one’s performance or functioning in the other role (e.g., family) is enhanced” (Wayne et al., 2004, p. 110). In recent years, positive aspects have received particular attention. Other similar concepts include positive spillover, enhancement, and enrichment (Tomida et al., 2019). Frone (2003) incorporated WFF as a component of WFB and pointed out the importance of addressing both negative (WFC) and positive (WFF) aspects. Notably, a low WFC does not necessarily mean a high WFF. Therefore, it is important to understand WFB to consider situations where WFC and WFF are both high. In this study, we use the concepts of WFC and WFF as indicators of WFB.

Based on these perspectives, Tomida et al. (2019) developed the WFB scale for middle-aged and older Japanese adults. The WFB scale includes four subscales assessing WFC and WFF in a bidirectional relationship between work and family roles (work-to-family and family-to-work). Two subscales evaluate the conflicts between work and family: work-to-family conflict (WF conflict) and family-to-work conflict (FW conflict), while the other two assess facilitation between work and family: work-to-family facilitation (WF facilitation) and family-to-work facilitation (FW facilitation). For example, the inability to help the family due to a busy work schedule indicates a WF conflict, whereas being absent from work because of family commitments indicates a FW conflict. Conversely, using work experience to solve problems at home is an example of WF facilitation, while the ability to solve problems at work because of experiences gained in the family is an example of FW facilitation.

We reason that possible classification of people based on levels of WFC and WFF would deepen our understanding of WFB. The person-oriented approach, which forms the basis of this way of typologizing WFB, is centered around the idea that “the totality gets its characteristics, features and properties from the interaction among the elements involved, not from the effect of each isolated part on the totality” (Bergman et al., 2003). In contrast, the component approach (Grzywacz and Carlson, 2007) has been widely used and considers WFC and WFF as components of the multidimensional WFB. In addition, this holistic perspective considers that the interaction of the elements involved differs from person to person. This typological view of WFB identifies and describes groups of individuals and is defined by similarities among multiple dimensions of interest. Therefore, it has been emphasized that conflict and facilitation do not exist in isolation and that their specific combination is important (Rantanen et al., 2011).

Frone (2003) presented a 4-fold taxonomy of WFB based on a literature review. The two primary dimensions of this taxonomy were the direction of influence between work and family roles (WF or FW) and its effect (conflict or facilitation). WFC and WFF are independent constructs. Optimal WFB is defined as having low WFC and high WFF. There have been only a few empirical studies based on this typological view of WFB, such as Demerouti and Geurts (2004), Grzywacz et al. (2008), Rantanen et al. (2011), and Rantanen et al. (2013). Based on the concept of Frone (2003), four types of WFB have been envisaged and tested, multiplying the highs and lows of the WFC by the highs and lows of the WFF, but no consistent and sufficient knowledge has been accumulated about this typological of WFB. Therefore, we used responses to the WFB scale (Tomida et al., 2019) to identify clusters that combine WFC and WFF. Furthermore, we explored the relationships between WFB clusters and participants’ demographic characteristics, work, family, and lifestyle factors, social support, and mental health.

Previous studies have identified variables that correlate with WFC. These include demographic characteristics, work, and family factors (Byron, 2005), and unhealthy lifestyle factors, such as sleep disturbances, unhealthy eating habits, smoking, and heavy alcohol consumption (e.g., Frone et al., 1996, 1997; Allen and Armstrong, 2006; Devine et al., 2006; Lallukka et al., 2010; Nelson et al., 2012; Leineweber et al., 2013). However, there are no empirical data on variables correlating with WFB clusters consisting of the combined effects of WFC and WFF. We focused on working hours and employment status, which are major work-related factors that have been associated with WFB in previous studies (e.g., Greenhaus and Beutell, 1985). However, caregiving roles, as a family factor, have rarely been investigated in previous WFB research (Neal et al., 2013). Therefore, in this study, family factors included middle-aged and older adults’ family roles—participation in household chores, childrearing, and caregiving for elderly family members. Lifestyle factors included sleep time, dietary behavior, current smoking status, and alcohol intake. Careful consideration of lifestyle factors could facilitate development of policies and strategies for maintaining work and family roles and for conducting appropriate interventions when needed.

In addition to these variables, social support and mental health are crucially related to WFB. A meta-analysis of social support studies demonstrated the benefits of social support within the work–family interface (French et al., 2018). Employees generally receive social support from supervisors, coworkers, and individuals outside of work, such as family and friends (Viswesvaran et al., 1999; Kossek et al., 2011). Receiving such support is negatively related to WFC (Adams et al., 1996; Kossek et al., 2011). Therefore, we hypothesized that the level of social support received would differ across WFB clusters.

Moreover, previous studies have suggested that WFC is associated with diverse mental health outcomes (for a review, see Allen et al., 2000) and is linked to mental health problems such as psychiatric disorders, depressive symptoms, and reduced life satisfaction (e.g., Frone et al., 1992, 1996; Higgins et al., 1992; Thomas and Ganster, 1995; Adams et al., 1996; Frone, 2000; Grzywacz, 2000; Shimazu et al., 2013). Therefore, WFC might account for differences in mental health, whereas the impact of WFF on mental health is less clear. We assessed the participants’ mental health, including their depressive symptoms and life satisfaction, and examined the associations between positive and negative mental health indicators and WFB clusters. We hypothesized that adequate WFB in middle-aged and older adults would be strongly associated with positive mental health.

The current study was conducted with middle-aged and older adults living in Japan. The study had two objectives: (a) to identify WFB clusters based on WFC and WFF in a stratified, randomly selected, community-based sample of working adults and (b) to explore the relationships between the WFB clusters and demographic characteristics, work factors, family factors, lifestyle factors, social support, and mental health.

## Materials and Methods

### Participants

The study sample consisted of 1,351 adults (788 males, 563 females; M_age_ = 54.82, SD_age_ = 9.86; age range = 40–85 years), selected from a pool of 2,330 participants in the seventh survey (July 2010 to July 2012) of the National Institute for Longevity Sciences-Longitudinal Study of Aging (NILS-LSA), who were working at the time of the survey and had no missing WFB scale data.

The NILS-LSA is a study of community-dwelling middle-aged and older adults (at least 40 years of age) in Japan (Shimokata et al., 2000). The first NILS-LSA survey was conducted between November 1997 and April 2000, and follow-up surveys were administered every 2 years until the seventh survey. The NILS-LSA is an invitation-type survey that uses a dynamic cohort methodology. In the first survey, people aged 40–79 years were randomly selected and stratified based on age and sex. Participants under 80 years of age who dropped out of the study during the follow-up surveys were replaced with individuals of the same age-decade group and sex. The participant pool was periodically replenished with new participants aged 40 years, to prevent cohort aging and maintain a survey population of approximately 2,300 people. The survey items related to WFB were first incorporated in the seventh survey. The questionnaires were mailed to the participants who completed them in their homes. A trained psychologist or psychology graduate student checked the completed questionnaires and re-administered questions with blank or unclear responses to minimize the possibility of missing data in the self-rated measures.

### Ethical Considerations

The protocol of this study was approved by the Ethics Committee on Human Research of the National Center for Geriatrics and Gerontology (no. 1350). All participants were informed about the specific details and significance of the study before enrollment. Written consent was obtained from all participants before they were enrolled in the study.

### Measures

Most of the scales used in this study were self-administered questionnaires. Details of the non-self-administered questionnaires are described below.

#### Work–Family Balance

WFB was assessed using the WFB Scale, based on the concepts of WFC and WFF (Tomida et al., 2019). In the WFB Scale, WFC is assessed using the 5-item “WF conflict” and the 5-item “FW conflict” subscales, while WFF is assessed by the 3-item “WF facilitation” and the 3-item “FW facilitation” subscales. Participants were asked to respond using a 5-point Likert scale ranging from 1 (disagree completely) to 5 (agree completely). Higher scores indicate more robust recognition of conflict or facilitation. In this study, Cronbach’s alpha coefficients for WF conflict, FW conflict, WF facilitation, and FW facilitation were 0.83, 0.85, 0.69, and 0.71, respectively.

#### Demographic Characteristics

Participants indicated their age (in years), sex (male or female), financial satisfaction (assessed on a 5-point scale, with higher scores indicating higher financial satisfaction), and educational attainment (number of years of post-primary education).

#### Work Factors

Participants indicated the number of hours they worked during a week and their employment status [regular employee, non-regular employee (part-time, contract employee, and temporary agency worker), or other (self-employed, agricultural/forestry/fishery worker, and other)].

#### Family Factors

Family factors included were the number of family members living with the participant, marital status (married or unmarried), household chores (no or yes), childrearing (no or yes; including children, grandchildren, and others), and care and assistance for family members with an illness or a disability (no, yes).

#### Lifestyle Factors

Lifestyle factors included dietary behavior, measured using the quantitative index for dietary diversity (QUANTIDD; Katanoda et al., 2006). The index was calculated using a three-day (two weekdays and one weekend day) dietary record maintained by participants by weighing food on a scale at home (1-kg kitchen scales; Sekisui Jushi, Tokyo, Japan) before cooking or by estimating portion sizes. The QUANTIDD ranges from 0 to 1, with lower scores indicating an unbalanced diet and higher scores indicating an equal distribution of each food group (see Otsuka et al., 2017, for more information). Alcohol intake (light: 0–19 ml, moderate: 20–59 ml, and heavy: 60 ml or more) was assessed as the average ethanol intake in the past year using the Food Frequency Questionnaire (FFQ; Shimizu et al., 1999), which is an interview survey. As a reference, the amount of alcohol contained in one medium-sized bottle of beer (500 ml) is approximately 20 ml of ethanol. Other lifestyle factors that were assessed included sleep time (hours per day) and current smoking status (no or yes).

#### Social Support

Social support was assessed using the Social Support Scale (Noguchi, 1991), which comprises 12 items assessing two specific social domains: family members and non-family members. Participants responded on a 4-point scale, and scores were calculated according to the method described by Noguchi (1991). Higher scores indicated higher perceived support. Cronbach’s alpha coefficient in this study was 0.81 for family members and 0.85 for non-family members.

#### Mental Health

Depression symptoms were assessed using the Center for Epidemiologic Studies Depression Scale (CES-D; Radloff, 1977; Shima et al., 1985). Participants responded to the CES-D using a 4-point scale, and scores were calculated such that higher scores indicated a higher level of depressive symptoms. Cronbach’s alpha coefficient of the scale in this study was 0.87.

Life satisfaction was evaluated using the Life Satisfaction Index K (LSI-K, Koyano, 1996), a 9-item questionnaire with a 2- or 3-point scale for each item. Scores were calculated according to the method described by Koyano (1996), such that higher scores indicated a higher level of life satisfaction. Cronbach’s alpha coefficient of the scale in this study was 0.64.

## Data Analyses

First, we calculated internal consistencies (Cronbach’s α), descriptive analyses, and correlations between the WFB scale and social support and mental health. Second, we performed cluster analysis of the WFB scale scores to extract the typology of WFB and computed ANOVA to test differences in WFB scale across clusters. Third, we computed ANOVA or chi-square analysis to test whether there was any statistically significant difference between demographic characteristics, family, work, and lifestyle factors across clusters. Finally, we computed ANOVAs to test whether there was any statistically significant difference between social support and mental health across clusters. All statistical analyses were conducted using the SAS version 9.3.

## Results

### Descriptive Analyses and Correlations of the Variables

Table 1 presents descriptive analyses and correlations of the variables. The pattern of correlations indicates weak to moderate positive significant correlations among the four WFB subscales, except for FW conflict and FW facilitation. Both, WF conflict and FW conflict, were negatively correlated with intra-family and extra-family social support, positively correlated with depression symptoms in mental health, and negatively correlated with life satisfaction. Conversely, WF facilitation and FW facilitation were positively correlated with social support within and outside the family, negatively correlated with depression symptoms, and positively correlated with life satisfaction.

**Table 1:** Means (*M*), standard deviation (*SD*), and intercorrelations between WFB, social support, and mental health.

	*M*	(*SD*)	1		2		3		4		5		6		7	
Work-Family Balance																
1. WF Conflict	2.41	(0.84)														
2. FW Conflict	1.83	(0.69)	0.58	^***^												
3. WF Facilitation	2.90	(0.83)	0.21	^***^	0.17	^***^										
4. FW Facilitation	3.22	(0.79)	0.14	^***^	0.04	*ns*	0.58	^***^								
Social Support																
5. Support from Family Members	35.19	(4.05)	−0.15	^***^	−0.20	^***^	0.18	^***^	0.36	^***^						
6. Support from Non-Family Members	32.01	(4.54)	−0.15	^***^	−0.06	^*^	0.14	^***^	0.19	^***^	0.48	^***^				
Mental Health																
7. Depression Symptoms	6.39	(6.45)	0.18	^***^	0.21	^***^	−0.12	^***^	−0.22	^***^	−0.28	^***^	−0.27	^***^		
8. Life Satisfaction	5.43	(1.97)	−0.17	^***^	−0.15	^***^	0.18	^***^	0.25	^***^	0.31	^***^	0.32	^***^	−0.58	^***^

WF Conflict, Work-to-Family Conflict; FW Conflict, Family-to-Work Conflict; WF Facilitation, Work-to-Family Facilitation; and FW Facilitation, Family-to-Work Facilitation. ^*^p < 0.05, ^***^p<0.001, and *ns* = not significant.

### Work–Family Balance Scale Cluster Analysis

**Figure 1:**
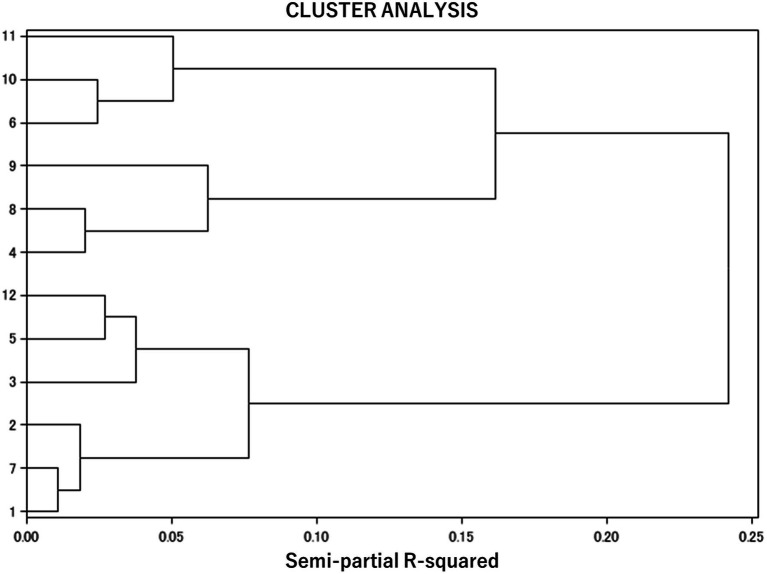
Diagram of cluster analysis.

A cluster analysis, using the Ward method, was conducted on the standardized scores of the four WFB subscales: WF conflict, FW conflict, WF facilitation, and FW facilitation. The cluster analysis using Ward’s method generated a dendrogram to estimate the number of clusters in the population and the differences between the clusters (Figure 1). According to the indices of pseudo-F, the semi-partial R-squared, and pseudo-t^2^, a good number of clusters was judged to be 3 or 4. Based on the dendrogram obtained, four-cluster configurations were selected, in order to avoid clusters with very small sample sizes and to facilitate the interpretability of the results. There were 315 participants in Cluster 1, 378 in Cluster 2, 407 in Cluster 3, and 251 in Cluster 4.

To verify the reproducibility of the four-class solution, we used a double-split, cross-validation procedure to examine the stability of the cluster solutions (Breckenridge, 2000) by splitting the sample randomly into two halves (samples A and B). Cluster analysis was conducted on the standardized scores of the four WFB subscales in the two groups. The agreement between these new clusters and the original clusters was determined using Cohen’s kappa (Subsample A, *κ* = 0.82; Subsample B, *κ* = 0.94). The guidelines provided by Fleiss (1981) characterize a kappa over 0.75 as excellent. The cluster solution with the highest kappa value is preferred because it is more stable and replicable. We further examined the exact reproducibility by sex (male and female) and age group (middle-aged adults are less than 60 years and older adults are aged 60 years or older). Convergence was indicated by sex (male, *κ* = 0.76: female, *κ* = 0.90) and age group (middle-aged, *κ* = 0.87: older, *κ* = 0.77), which justified our approach of conducting cluster analysis with the full sample.

**Figure 2:**
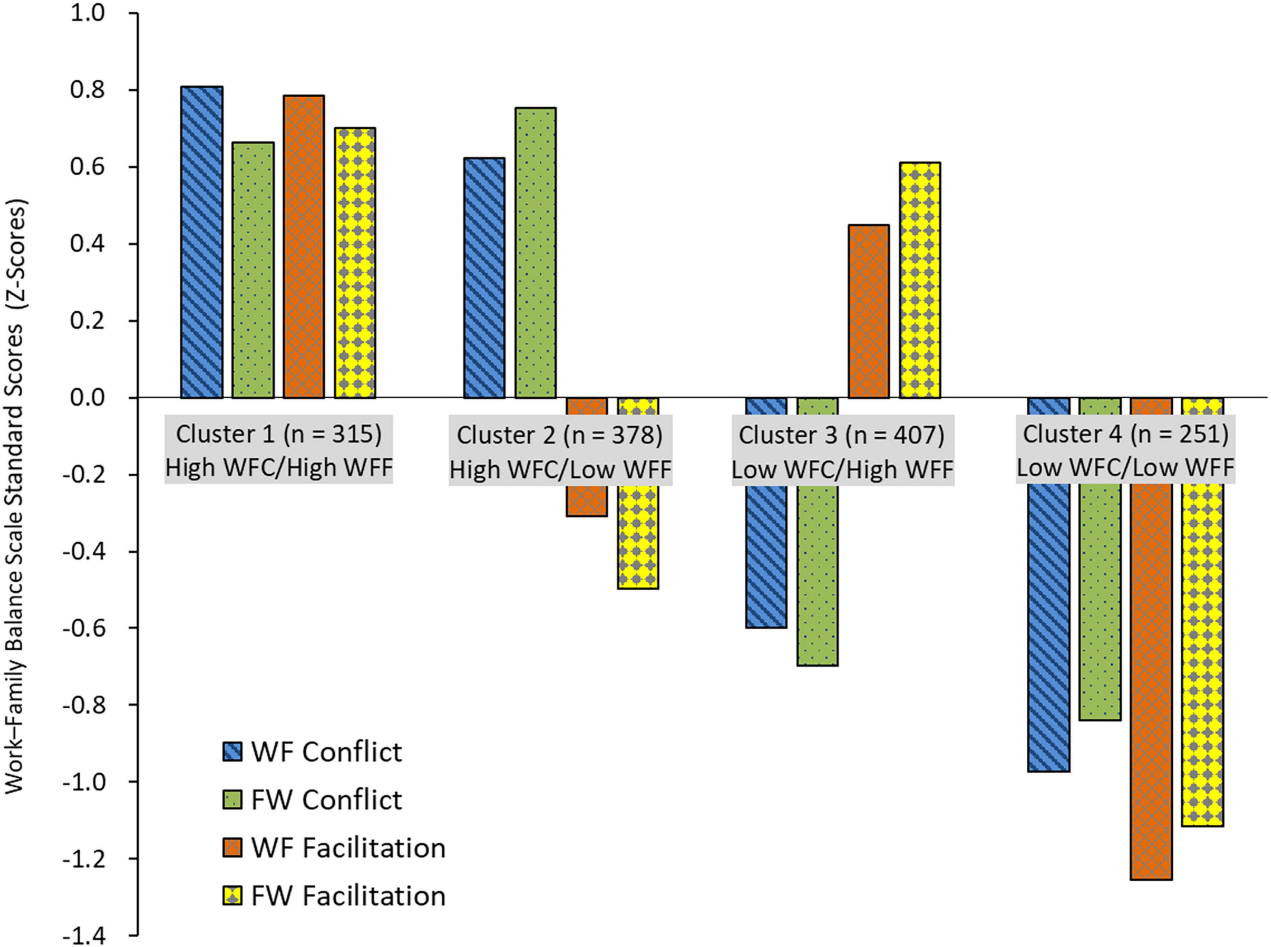
Four clusters and work–family balance scale scores. WFC, Work–Family Conflict; WFF, Work-Family Facilitation; WF Conflict, Work-to-Family Conflict; FW Conflict, Family-to-Work Conflict; WF Facilitation, Work-to-Family Facilitation; and FW Facilitation, Family-to-Work Facilitation.

An ANOVA was conducted with the four clusters as independent variables and the four WFB subscales as dependent variables to identify characteristics of each cluster. ANOVA indicated significant differences between the clusters. A *post-hoc* Tukey–Kramer analysis revealed the following orders for WF conflict (*F* = 537.80, *p* < 0.001): Cluster 1 > 2 > 3 > 4 (all *p* < 0.05; hereafter, the same test was performed); FW conflict (*F* = 534.45, *p* < 0.001): Cluster 1, 2 > 3 > 4; WF facilitation (*F* = 493.69, *p* < 0.001): Cluster 1 > 3 > 2 > 4; and FW facilitation (*F* = 502.58, *p* < 0.001): Cluster 1, 3 > 2 > 4. Cluster 1 had high WFC and WFF, Cluster 2 had high WFC and WFF, Cluster 3 had low WFC and WFF, and Cluster 4 had low WFC and WFF (Figure 2).

### Correlates of the Work–Family Balance Clusters

Demographic characteristics, family, work, and lifestyle factors were similarly analyzed to identify the attributes of the four clusters. ANOVA or chi-square analysis was conducted with the four clusters, and the results indicated significant differences between all factors, except educational attainment, across the clusters (Table 2).

**Table 2:** Demographic characteristics and work, family and lifestyle factors across work–family balance clusters.

		Cluster 1			Cluster 2			Cluster 3			Cluster 4			*F*/*χ*^2^ value	*p*	Tukey–Kramer method
	High-WFC/High-WFF			High-WFC/Low-WFF			Low-WFC/High-WFF			Low-WFC/Low-WFF						
**Demographic Characteristics**
**Age (years)^a^**		55.80	10.20		53.15	8.80		55.15	9.90		55.55	10.40		5.34	**	1, 3, 4 > 2
**Sex^b^**
Male		178	(13.18)		209	(15.47)		261	(19.32)	△	140	(10.36)		8.17	*	
Female		137	(10.14)		169	(12.51)		146	(10.81)	▼	111	(8.22)				
**Financial Satisfaction^a^**		3.25	1.10		2.92	1.10		3.43	1.10		3.08	1.10		15.05	^***^	1, 3 > 2, 3 > 4
Educational Attainment (years)^a^		13.60	2.60		13.55	2.30		13.50	2.70		13.32	2.50		0.62	*ns*	
**Work Factors**
**Working Hours (hours/week)^a^**		39.64	16.60		38.60	15.60		35.78	15.30		32.25	14.90		12.72	^***^	2 > 4, 1 > 3 > 4
**Employment Status^b^**
Regular Employee		128	(9.47)	▼	190	(14.06)	△	201	(14.88)		102	(7.6)		67.12	^***^	
Non-Regular Employee		88	(6.51)	▼	124	(9.18)		148	(10.95)		123	(9.10)	△			
Other		99	(7.33)	△	64	(4.74)		58	(4.29)	▼	26	(1.92)	▼			
**Family Factors**
**Family Living Together (*n*)^a^**		2.54	1.50		2.63	1.60		2.36	1.50		2.30	1.50		3.47	*	2 > 4
**Marital Status^b^**
Married		288	(21.32)	△	328	(24.28)		355	(26.28)		206	(15.25)	▼	10.96	*	
Unmarried		27	(2.00)	▼	50	(3.70)		52	(3.85)		45	(3.33)	△			
**Household Chores^b^**
No		69	(5.11)		77	(5.70)	▼	123	(9.10)	△	70	(5.18)		12.93	**	
Yes		246	(18.21)		301	(22.28)	△	284	(21.02)	▼	181	(13.40)				
**Child-Rearing^b^**
No		250	(18.50)		282	(20.87)	▼	335	(24.80)		204	(15.10)		7.92	*	
Yes		65	(4.81)		96	(7.11)	△	72	(5.33)		47	(3.48)				
**Care and Assistance^b^**
No		251	(18.58)	▼	317	(23.46)	▼	378	(27.98)	△	227	(16.80)		32.83	^***^	
Yes		64	(4.74)	△	61	(4.52)	△	29	(2.15)	▼	24	(1.78)				
**Lifestyle Factors**
**Sleeping time (hour/day)^a^**		6.88	1.06		6.75	1.00		6.94	1.04		6.98	0.98		3.30	^*^	3, 4 > 2
**Dietary Behavior (Dietary Diversity)^a^**		0.88	0.05		0.87	0.05		0.88	0.04		0.87	0.06		3.85	^**^	1, 3 > 2
**Current Smoking^b^**
No		279	(20.65)	△	308	(22.80)		344	(25.46)		203	(15.03)		8.56	^*^	
Yes		36	(2.66)	▼	70	(5.18)		63	(4.66)		48	(3.55)				
**Alcohol Intake^b^**
Light Drinking: <20 ml/day		249	(18.56)	△	277	(20.64)		266	(19.82)	▼	178	(13.27)		23.52	^**^	
Moderate Drinking: 20–60 ml/day		48	(3.58)	▼	64	(4.77)		106	(7.90)	△	45	(3.35)				
Heavy Drinking: ≥60 ml/day		17	(1.27)	▼	33	(2.46)		32	(2.38)		27	(2.01)				

WFC, Work–Family Conflict; WFF, Work-Family Facilitation. If a significant difference was observed by the chi-square test, a residual analysis was performed. A significantly lower value is shown as ▼, and a significantly greater value as △ (p < 0.05). If a significant difference was observed by an analysis of variance (ANOVA), multiple comparisons (Tukey–Kramer method) was performed (p < 0.05). N ranges from 1,341 to 1,354 due to missing values, ^a^Mean, SD (Standard Deviations). ^b^Number, (%), ^*^p < 0.05, ^**^p < 0.01, and ^***^p < 0.001.

#### Demographic Characteristics

The main effect of clusters was significant for age and financial satisfaction. The significant differences indicated by a multiple comparison test revealed the following orders for age (*F* = 5.34, *p* < 0.01): Cluster 1, 3, 4 > 2 and financial satisfaction (*F* = 15.05, *p* < 0.001): Cluster 1, 3 > 2, and Cluster 3 > 4. Educational attainment was not associated with any of the clusters (*F* = 0.62, n.s.). A chi-square analysis with demographic characteristics as the categorical variables indicated significant differences in frequency by sex (*χ*^2^ = 8.17, *p* < 0.05). Residual analyses were performed for categorical variables with statistically significant chi-square values. The results are shown in Table 2 (all *p* < 0.05).

#### Work Factors

The main effect of clusters was significant for working hours. A *post-hoc* Tukey–Kramer analysis revealed the following orders for working hours (*F* = 12.72, *p* < 0.001): Cluster 2 > 4 and Cluster 1 > 3 > 4. A chi-square analysis, with work factors as the categorical variables, indicated significant differences in employment status frequencies (*χ*^2^ = 67.12, *p* < 0.001).

#### Family Factors

The main effect of clusters was significant for living with the family. Post-hoc Tukey–Kramer analysis revealed the following order for living with family (*F* = 3.47, *p* < 0.05): Cluster 2 > 4. A chi-square analysis with family factors as the categorical variables indicated significant differences in the frequencies of marital status (*χ*^2^ = 10.96, p < 0.05), household chores (*χ*^2^ = 12.93, *p* < 0.01), childrearing (*χ*^2^ = 7.92, *p* < 0.05), and care and assistance (*χ*^2^ = 32.83, *p* < 0.001).

#### Lifestyle Factors

The main effect of clusters was significant for sleep time. Post-hoc Tukey–Kramer analysis revealed the following orders for sleeping time (*F* = 3.30, *p* < 0.05): Cluster 3, 4 > 2 and dietary behavior (*F* = 3.85, *p* < 0.01): Cluster 1, 3 > 2. A chi-square analysis with lifestyle factors as the categorical variables indicated significant differences in the frequencies of current smoking status (*χ*^2^ = 8.56, *p* < 0.05) and alcohol intake (*χ*^2^ = 23.52, *p* < 0.01).

### Social Support and Mental Health Across the Work–Family Balance Clusters

ANOVA was conducted with the four clusters as the independent variables, and social support and mental health as the dependent variables (Table 3).

**Table 3:** Social support and mental health across work–family balance clusters.

	Cluster 1		Cluster 2		Cluster 3		Cluster 4		*F* value	*p*	Tukey–Kramer method	HighWFC/HighWFF		HighWFC/LowWFF		LowWFC/HighWFF		LowWFC/LowWFF		*M*	(*SD*)	*M*	(*SD*)	*M*	(*SD*)	*M*	(*SD*)
Social Support											
Support from Family Members	35.56	(3.47)	33.47	(3.58)	36.85	(4.04)	34.65	(4.28)	53.18	^***^	3 > 1 > 4 > 2
Support from Non-Family Members	32.30	(4.18)	30.78	(4.41)	33.20	(4.46)	31.58	(4.77)	20.55	^***^	3 > 1 > 2, 3 > 4
Mental Health											
Depression Symptoms	6.53	(6.31)	8.35	(6.94)	4.17	(4.99)	6.88	(6.91)	30.12	^***^	2 > 1, 4 > 3
Life Satisfaction	5.50	(1.94)	4.84	(1.94)	6.10	(1.76)	5.16	(2.07)	30.23	^***^	3 > 1 > 2, 3 > 4

WFC, Work–Family Conflict; WFF, Work-Family Facilitation. Values in the table are *Means* and *SD* (*Standard Deviations*). If a significant difference was observed by an analysis of variance (ANOVA), multiple comparisons (Tukey–Kramer method) was performed (*p* < 0.05). ^***^p<0.001.

#### Social Support

The main effect of the clusters was significant for social support. *Post-hoc* Tukey–Kramer analysis revealed the following order for social support from family members (*F* = 53.18, *p* < 0.001): Cluster 3 > 1 > 4 > 2 and social support from non-family members (*F* = 20.55, *p* < 0.001): Cluster 3 > 1 > 2 and Cluster 3 > 4 (all *p* < 0.05).

#### Mental Health

The main effect of clusters was significant for mental health. A *post-hoc* Tukey–Kramer analysis revealed the following order for symptoms of depression (*F* = 30.12, *p* < 0.001): Cluster 2 > 1, 4 > 3 and life satisfaction (*F* = 30.23, *p* < 0.001): Cluster 3 > 1 > 2 and Cluster 3 > 4 (all *p* < 0.05).

These differences in social support and mental health across the clusters did not change even after controlling for demographic variables such as age, sex, financial satisfaction, educational attainment, working hours, employment status, and marital status (results not shown).

## Discussion

This study identified the typology of WFB using cohort data collected from middle-aged and older Japanese adults and described the four typologies in terms of their associations with demographic variables, work factors, family factors, lifestyle factors, social support, and mental health.

### Work–Family Balance Clusters of Middle–Aged and Older Adults

The four clusters we extracted through cluster analysis, consisted of different combinations of the positive and negative aspects of the WFB. Moreover, they confirmed the conceptual framework posited by Frone (2003), who claimed that WFC and WFF are independent constructs. We identified the following four clusters based on WFF and WFC: Cluster 1 (high WFC, high WFF), Cluster 2 (high WFC and low WFF), Cluster 3 (low WFC and high WFF), and Cluster 4 (low WFC and low WFF).

The reproducibility of the four-cluster solution was examined using a double-split cross-validation procedure. We confirmed that the same WFB profiles appeared in both halves of the two-part random, sex-stratified, and age-stratified data. Moreover, the kappa values across the subsamples provided substantial evidence of the stability of the four-cluster solution. In this study, four clusters based on the person-centered approach were extracted, and the degree of agreement with the typology that assigns subjects based on the researcher’s predetermined criterion (median criterion) was also verified. The four typologies were created based on the median criterion of WFC and WFF (data not shown). We calculated Cohen’s kappa (*κ* = 0.23) to confirm the degree of agreement between the four clusters extracted in this study and the four types based on the median criterion, and found a certain degree of agreement (Landis and Koch, 1977). In this study, cluster analysis was used to more accurately identify the types of WFB that naturally exist in middle-aged and older Japanese, and the fact that the extracted types showed a certain degree of agreement with the types assumed by the researchers is considered to guarantee the characteristics of the clusters extracted in this study.

### Correlates of the Work–Family Balance Clusters

We examined the relationship between the four clusters and certain variables, including interdisciplinary indicators closely related to WFB, to identify the clusters’ characteristics. The results indicated that the four groups were related to distinct demographic patterns, family, work, and lifestyle factors.

Cluster 1 (high WFC and high WFF) included a high proportion of married people and people with a high degree of financial satisfaction, long working hours, and employment other than regular or non-regular employment. However, they were burdened by assisting others. The lifestyle in this cluster was characterized by good dietary behavior, light drinking, and no smoking. Individuals in Cluster 1 were more likely to feel the strain of work and family roles, while also experiencing specific positive aspects of a balanced life.

Cluster 2 (high WFC and low WFF) included relatively young people who tended to work long hours. This cluster also included a high proportion of people with regular employment and those living with family members. The cluster also included many people with a heavy burden of household chores, childrearing, and assisting others. Consequently, short sleep hours and suboptimal eating behaviors hampered their lifestyles. Cluster 2 comprises hard-working employees struggling to manage their work and family; they seem to face many difficulties in achieving an appropriate WFB.

Cluster 3 (low WFC and high WFF) was the most desirable and was associated with healthy lifestyle factors. This cluster was characterized by a large number of men, short working hours, and few people with employment status of “other,” such as self-employed or agricultural/forestry/fishery workers. Cluster 3 also included people with high financial satisfaction, few household chores, and a relatively light burden in terms of assisting others. Moreover, their lifestyles included long sleep duration, good eating habits, and moderate drinking habits. Overall, Cluster 3 individuals seem to enjoy many positive and relatively few negative aspects of WFB.

Finally, Cluster 4 (low WFC and low WFF) included many participants with short working hours and non-regular employment, who were unmarried, and who lived with only a few family members. Lifestyle factors included long sleep duration. Overall, people in this cluster were characterized by low levels of work and family commitment.

We observed no significant differences in educational attainment across the clusters. Additionally, there were only a few heavy alcohol consumers in all the clusters. Only relatively healthy and well-educated people participated in this study because an institutional sample was used. We also found no association between alcohol consumption and WFB, contrary to previous studies (Frone et al., 1996, 1997; Lallukka et al., 2010; Leineweber et al., 2013). The participants in this study might have had the luxury to adjust their work schedule to spare some time to participate in the survey and held a high level of interest in personal health. Moreover, this study might have been influenced by the “healthy worker survival effect” bias (Arrighi and Hertz-Picciotto, 1994), because unhealthy workers might have dropped out of this cohort study.

### Social Support and Mental Health Across the Work–Family Balance Clusters

The results indicate significant cluster differences for each source of social support. The participants in Cluster 3 experienced high social support from family and non-family members. Cluster 1 also comprised participants with relatively high levels of social support. However, participants in Clusters 2 and 4 received low social support from either family or non-family members. These findings highlight the need for middle-aged and older adults to have appropriate social support from both family and non-family members to develop a healthy WFB.

We also identified significant cluster differences in mental health. Compared to Clusters 1, 2, and 4, the participants in Cluster 3 had relatively better mental health, experienced the fewest depressive symptoms, and had high life satisfaction. Conversely, the participants in Cluster 2 (high WFC and low WFF) had many negative experiences, such as a high role load at home, an unhealthy lifestyle, and the poorest mental health, which was combined with only a few positive experiences. Participants in Cluster 1 (high WFC and high WFF) and 4 (low WFC and low WFF) had more negative experiences and poorer mental health than those in Cluster 3. In particular, Cluster 1 participants experienced both positive and negative aspects in balancing their home and work life, but the negative aspects might have had a more substantial impact on their mental health.

These results suggest that WFB can be classified into four types in terms of low vs. high levels of WFC and WFF, each characterized by demographic characteristics, family, work, and lifestyle factors. Moreover, there were significant differences in social support and mental health across the groups. A review by Greenhaus and Beutell (1985) suggested that social support has a mediating or buffering effect on the relationship between WFC and mental health. Additionally, Viswesvaran et al. (1999) indicated that the social support received by individuals, regardless of whether it is in family, work, or leisure domains, can have a positive influence on workers’ mental health and life satisfaction. This study identified an association between WFB clusters, social support, and mental health. However, this study did not examine causality in these relations. This aspect needs to be explored in future research.

### Conclusion, Future Considerations, and Limitations

We used variables related to the WFB of middle-aged and older adults, including WFF and WFC, and classified the participants into clusters. Then, we identified each cluster’s features based on its relationships with variables such as demographic characteristics, work, family, lifestyle factors, social support, and mental health. Despite the vagueness of the WFB concept, the cluster-based approach used in this study successfully identified low WFC and high WFF (Cluster 3) as ideal for maintaining an adequate WFB. From a theoretical perspective, the four-dimensional typology of WFB detected here supported four types of prior research based on Frone (2003), who presented a 4-fold taxonomy of WFB (Demerouti and Geurts, 2004; Grzywacz et al., 2008; Rantanen et al., 2011).

The study also highlights the relationship between lifestyle factors, social support, mental health, and WFB. Inadequate WFB was correlated with many risky lifestyle factors. Conversely, perceived social support could be a useful resource for maintaining a healthy WFB. The typologies of WFB have crucial implications for mental health. As illustrated in this study, research that investigates WFC and WFF together, would promote our understanding of how lifestyle factors differ across different degrees of WFB and how social support and mental health within a given lifestyle could affect levels of WFB. Therefore, it might be possible to improve mental health by eliminating lifestyle factors unsuitable for maintaining WFB and providing appropriate social support. These findings are significant for understanding the theoretical and practical aspects of the work–family interface.

A limitation of this study lies in the nature of this descriptive cross-sectional study. We described and compared the levels of a series of variables across different WFB clusters and paid little attention to the causal nature among those variables, that is, the possibility that people who are less depressed are more likely than others to maintain a sufficient WFB. Longitudinal research is needed to explore this possibility. Moreover, longitudinal studies are needed to better understand the relationships among WFB, social support, and mental health.

Another limitation of this study is that the data on which it is based were collected between 2010 and 2012 and are not the most recent available. In April 2021, the Law Concerning Stabilization of Employment of Older Persons was amended to require companies to lower the mandatory retirement age from 65 to 70, or to continue employment until 70, or to abolish the mandatory retirement age. One of the key issues relating to the ageing of the workforce in Japanese society is the possibility that issues such as personal health and the care of family members will become increasingly apparent in the future, as will the sustainability of work. In particular, the above characteristics were found in cluster 2 (high WFC/low WFF), which is slightly younger in age than the other clusters. It is also considered to be the core of the working generation but shows a high work–family load, with significant care giving, child rearing, and long working hours. In addition, cluster 2 is characterized by low levels of social support received and therefore low levels of mental health. There is an urgent need to establish the support resources required for those in cluster 2.

The third challenge is to confirm the replicability of the clusters found in this study with different data and longitudinal data. More advanced statistical methods, such as Latent Profile Analysis (LPA) used by Rantanen et al. (2013), should allow us to statistically confirm the appropriateness of the classification of the clusters.

Japan leads the world in population aging and can serve as an appropriate model for other countries following the same demographic path. Despite the limitations mentioned above, we believe that this study contributes to the typology of WFB in middle-aged and older Japanese adults by considering the role of WFC and WFF. We hope that this study will follow a series of cross-cultural research to expand the horizons of the WFB literature.

## Data Availability Statement

The datasets presented in this article are not readily available. The data that support the findings of this study are available from the National Institute for Longevity Sciences-Longitudinal Study of Aging (NILS-LSA). However, restrictions apply to the availability of these data, which were used under license for the current study, and are, thus, not publicly available. Data can be made available by the authors upon reasonable request and with permission from the NILS-LSA. Requests to access the datasets should be directed to NILS-LSA office, nilslsa@ncgg.go.jp.

## Ethics Statement

The studies involving human participants were reviewed and approved by the Ethics Committee on Human Research of the National Center for Geriatrics and Gerontology. The patients/participants provided their written informed consent to participate in this study.

## Author Contributions

MT drafted the manuscript and took responsibility for the accuracy of the data analysis. MT, YN, CT, TN, and RO contributed to the data collection and statistical analyses. HS and FA contributed to the study concept and design of the NILS-LSA. YN, CT, TN, RO, HS, and FA interpreted the results. MT and RO obtained funding. All authors contributed to the interpretation of the data and have approved the final version of the manuscript before submission and publication.

## Funding

This work was supported in part by the Japan Society for the Promotion of Science KAKENHI (grant numbers JP26-3405 and JP17K13958) and the Research Funding for Longevity Sciences from the National Center for Geriatrics and Gerontology, Japan (grant numbers 21-18).

## Conflict of Interest

The authors declare that the research was conducted in the absence of any commercial or financial relationships that could be construed as a potential conflict of interest.

## Publisher’s Note

All claims expressed in this article are solely those of the authors and do not necessarily represent those of their affiliated organizations, or those of the publisher, the editors and the reviewers. Any product that may be evaluated in this article, or claim that may be made by its manufacturer, is not guaranteed or endorsed by the publisher.
